# Hemodynamic and anti-inflammatory effects of early esmolol use in hyperkinetic septic shock: a pilot study

**DOI:** 10.1186/s13054-020-03445-w

**Published:** 2021-01-07

**Authors:** Bruno Levy, Caroline Fritz, Caroline Piona, Kevin Duarte, Andrea Morelli, Philippe Guerci, Antoine Kimmoun, Nicolas Girerd

**Affiliations:** 1 Service de Médecine Intensive Et Réanimation Brabois, CHRU Nancy, Pôle Cardio-Médico-Chirurgical, 54511 Vandœuvre-lès-Nancy, France; 2 INSERM U1116, Faculté de Médecine, 54511 Vandœuvre-lès-Nancy, France; 3 Université de Lorraine, 54000 Nancy, France; 4 INSERM CIC1433, Nancy University Hospital, 54000 Nancy, France; 5 Department of Internal Clinical, Anesthesiological and Cardiovascular Sciences, University of Rome, La Sapienza, Rome, Italy; 6 Department of Anesthesiology and Intensive Care Medicine, University Hospital of Nancy, 54511 Vandœuvre-lès-Nancy, France; 7 Medical Intensive Care Unit, University Hospital of Nancy, Brabois, Rue du Morvan, 54500 Vandœuvre-lès-Nancy, France

**Keywords:** Septic shock, Inflammation, Norepinephrine, Beta-blockers

## Abstract

**Background:**

Several studies have shown that heart rate control with selective beta-1 blockers in septic shock is safe. In these trials, esmolol was administered 24 h after onset of septic shock in patients who remained tachycardic. While an earlier use of beta-blockers might be beneficial, such use remains challenging due to the difficulty in distinguishing between compensatory and non-compensatory tachycardia. Therefore, the Esmosepsis study was designed to study the effects of esmolol aimed at reducing the heart rate by 20% after the initial resuscitation process in hyperkinetic septic shock patients on (1) cardiac index and (2) systemic and regional hemodynamics as well as inflammatory patterns.

**Methods:**

Nine consecutive stabilized tachycardic hyperkinetic septic shock patients treated with norepinephrine for a minimum of 6 h were included. Esmolol was infused during 6 h in order to decrease the heart rate by 20%. The following data were recorded at hours H0 (before esmolol administration), H1–H6 (esmolol administration) and 1 h after esmolol cessation (H7): systolic arterial pressure, diastolic arterial pressure, mean arterial pressure, central venous pressure, heart rate, PICCO transpulmonary thermodilution, sublingual and musculo-cutaneous microcirculation, indocyanine green clearance and echocardiographic parameters, diuresis, lactate, and arterial and venous blood gases.

**Results:**

Esmolol was infused 9 (6.4–11.6) hours after norepinephrine introduction. Esmolol was ceased early in 3 out of 9 patients due to a marked increase in norepinephrine requirement associated with a picture of persistent cardiac failure at the lowest esmolol dose. For the global group, during esmolol infusion, norepinephrine infusion increased from 0.49 (0.34–0.83) to 0.78 (0.3–1.11) µg/min/kg. The use of esmolol was associated with a significant decrease in heart rate from 115 (110–125) to 100 (92–103) beats/min and a decrease in cardiac index from 4.2 (3.1–4.4) to 2.9 (2.5–3.7) l/min/m^−2^. Indexed stroke volume remained unchanged. Cardiac function index and global ejection fraction also markedly decreased. Using echocardiography, systolic, diastolic as well as left and right ventricular function parameters worsened. After esmolol cessation, all parameters returned to baseline values. Lactate and microcirculatory parameters did not change while the majority of pro-inflammatory proteins decreased in all patients.

**Conclusion:**

In the very early phase of septic shock, heart rate reduction using fast esmolol titration is associated with an increased risk of hypotension and decreased cardiac index despite maintained adequate tissue perfusion (NCT02068287).

## Introduction

The use of high-dose norepinephrine (NE) and concurrent tachycardia are associated with poor outcomes in septic shock (1). Despite the fact that the majority of the trials displayed beneficial results for selective and very short half-life beta 1-blocker use in patients with septic shock (2, 3), there is still a need for a large RCT (4). The majority of these trials used esmolol 24 h after onset of septic shock in patients who remained tachycardic (5, 6). It remains unknown whether early esmolol treatment might yield better results. (7). On the other hand, an early use of beta-blockers might be difficult due to the challenge in distinguishing between the compensatory or non-compensatory origin of tachycardia (8). Indeed, in the very early phase of septic shock, the combinational effects of inflammatory mediators, fluid loading and increasing afterload with NE may worsen myocardial contractility. In this condition, tachycardia is a crucial compensatory response. On the other hand, the so-called dysautonomic tachycardia is associated with a concomitant reduction in diastolic time, in ventricular filling and, ultimately, in cardiac index. In this instance, the use of selective beta-1 blockers may prove beneficial. This latter point is highlighted by rodent studies using beta-blockers that show either a maintenance or an increase in cardiac output despite the decrease in heart rate. In human septic shock treated with beta-blockers, depending on the studies, cardiac index either decreased or remained stable but with good tolerance with regard to tissue oxygenation parameters. Therefore, the Esmosepsis study was designed to study the effects of esmolol aimed at reducing the heart rate by 20% after the initial resuscitation process in hyperkinetic septic shock on (1) cardiac index and (2) systemic and regional hemodynamics as well as inflammatory patterns.

## Materials and methods

### Study design and oversight

The University Hospital Center in Nancy (France) designed and sponsored the trial. Trial administration, data management and statistical analysis were performed by the sponsor. The executive committee had unrestricted access to the data, and the authors analyzed the data and prepared the manuscript. This single-center pilot phase 2 open-label study was conducted between December 2013 (first inclusion) and March 2017 (last follow-up) in two French Intensive Care Units (ICU) in Nancy France (NCT02068287, Esmolol Effects on Heart and Inflammation in Septic Shock (ESMOSEPSIS)). The study received the approval of the Nancy Hospital Institutional Review Board (Board (CPP 12.12.03, EudraCT: 2012-004532-32). Written informed consent was obtained from the patients or their closest relatives. The trial was overseen by an independent data safety monitoring board.

### Study population

Patients with septic shock according to the 2012 criteria (9) were eligible if they were older than 18 years of age and fulfilled the following criteria: (1) inclusion as soon as possible after at least six hours of norepinephrine administration and fluid optimization using dynamic parameters; (2) a cardiac index higher than 3 l/min/m^2^ and (3) a heart rate higher than 100 beats/min.

Exclusion criteria were shock of other origin, severe septic cardiomyopathy and history of severe asthma, patients without social assurance, and adult patients under legal protection.

### Study treatments and protocol

Norepinephrine and esmolol doses are expressed in μg/kg/min. In instances of a decrease in MAP during esmolol infusion, norepinephrine doses were increased by 0.02 μg/kg/min (or higher in emergency cases). The targeted MAP was 65–70 mmHg.

#### Esmolol administration

Esmolol (Brevibloc^R^, Baxter, Saint-Quentin-en-Yvelines, France) was initiated at 7.5 µg/kg/min implemented in 5-min increments until the effects on heart rate were reached. Treatment duration with esmolol was 6 h maximum. The maximum maintenance dose was 200 µg/kg/min. If the goal of 20% heart rate reduction was not achieved with the maximum maintenance dose of 200 µg/kg/min, the patient remained treated with this dose until the end of the 6 h and was evaluated as such. In cases of a greater than 20% decrease in cardiac index during esmolol infusion, its dosage was reduced by 25 µg/kg/min until restoration of the cardiac index to the safety target. A hemodynamic evaluation was performed prior to each dose modification. If a preload dependency was identified, the patient underwent vascular filling using 250 ml saline infused in 10 min until resolution. Fluid optimization was performed using passive leg raising and PICCO transpulmonary thermodilution and pulse contour analysis as previously described (10).

### Monitoring

#### Transpulmonary thermodilution and pulse contour analysis

All patients had an internal jugular vein catheter and a thermistor-tipped arterial catheter (PV2024 Pulsion Medical Systems, Munich, Germany) inserted in the femoral artery and connected to the PiCCO2 device. Three cold boluses were administered when performing transpulmonary thermodilution. This allowed measuring cardiac index (CI) (through transpulmonary thermodilution and pulse contour analysis), global end-diastolic volume and extravascular lung water (both through transpulmonary thermodilution). Global ejection fraction, index stroke volume and cardiac function index were calculated according to standard formulas.

### Sidestream dark field (SDF) methodology

Microcirculation was assessed using a Sidestream Dark Field (SDF) imaging device (Microscan®, MicroVision Medical, Amsterdam, Netherlands). An automated Vascular Analysis software (AVA 3.0 Software, MicroVision Medical, Amsterdam, Netherlands) was used for image analysis according to current guidelines (11). Five sublingual areas were focused for at least > 20 s at each measurement. The following parameters were collected: total and perfused vessel density (TVD, PVD), proportion of perfused vessels (PPV) and microvascular flow index (MFI).

### Plasma disappearance rate of indocyanine green (ICG-PDR)

The plasma disappearance rate of indocyanine green (ICG-PDR) was used as a dynamic test for the assessment of liver function and global hepatosplanchnic blood flow (12). The ICG-PDR was assessed with a noninvasive liver function monitoring system (LiMon, Pulsion Medical Systems, Munich, Germany). Each patient received an ICG finger clip connected to the liver function monitor. A dose of 0.25 mg/kg of ICG was injected through a central venous catheter.

### Near infrared spectroscopy (NIRS)

Tissue oxygen saturation (StO2) was measured by a tissue spectrometer (InSpectra Model 325, Hutchinson Technology, Hutchinson, Minn.) through reflectance mode probes to measure scattering light reflected at a distance from where the light is transmitted into the tissue. The NIRS probe was placed on the skin of the thenar eminence and a sphygmomanometer cuff was wrapped around the arm over the brachial artery. After a 3-min NIRS signal stabilization period, arterial inflow was stopped by inflating the cuff to 50 mmHg above the systolic arterial pressure. When StO_2_ was under 40%, cuff pressure was released, and StO_2_ was recorded continuously for another 3 min period (reperfusion period). Baseline StO_2_ was recorded prior to the ischemic period, the lowest StO_2_ recorded at the end of the ischemic period, and the highest StO_2_ recorded during the reperfusion phase. The slope of the increase in StO2 obtained by the regression line of the first five StO_2_ values (14 s) during the reperfusion phase following the ischemic period (StO_2_ resaturation slope, expressed in % per second) as well as the difference between the maximum StO_2_ value during the hyperemic phase and the baseline StO_2_ (StO_2_ overshoot) were calculated. The StO_2_ desaturation slope (expressed in % over time) during the ischemic phase was also calculated.

### Echocardiography

Echocardiography was performed by an experimented investigator using a Vivid E90 (GE Healthcare) in which the following parameters were recorded: velocity time integral (VTI), left ventricular ejection fraction (LVEF), tricuspid annular plane systolic excursion (TAPSE), transmitral *E*/*A* ratio (E/A), *e*′ velocity (average and absolute value of septal and lateral side) and systolic (*s*′) velocity of lateral tricuspid annulus by pulsed tissue Doppler.

### Olink® Inflammation reagent kit

Plasma samples were collected at H0 and H6 and stored at the study sites at − 20 °C, followed by storage at − 80 °C at the central laboratory. Olink® Inflammation is a reagent kit measuring 92 inflammation-related human protein biomarkers simultaneously. Measurement details can be found at https://www.olink.com/content/uploads/2019/04/Olink-Inflammation-Validation-Data-v3.0.pdf.

### Measured variables

The following data were recorded at hours H0 (before esmolol administration), H1 to H6 (esmolol administration) and 1 h after esmolol cessation (H7): systolic arterial pressure (SAP), diastolic arterial pressure (DAP), mean arterial pressure (MAP), central venous pressure, heart rate, PICCO transpulmonary thermodilution, NIRS, SDF, Limon and echocardiography parameters, diuresis, lactate, and arterial and venous blood gases. Indexed oxygen delivery (DO2i) and indexed oxygen consumption (VO_2_i) were calculated using standard formulas.

### Outcomes

Primary Outcome:

Since the decrease in heart rate directly influences cardiac index, the change in CI was selected as the primary outcome variable during the entire administration period and one hour after esmolol cessation.

Secondary outcome measures:

1. Effects on vasopressor requirement (amount of norepinephrine infused in microgram/kg) during esmolol administration: recording of each change in vasopressor dosage to maintain a mean arterial pressure at 70 mmHg during the entire esmolol administration period (H0 to H6) and one hour after esmolol cessation (H7).
2. Microcirculatory and regional circulation effects of esmolol in septic shock patients. NIRS (near-infrared spectroscopy), SDF (Sidestream Dark Field imaging) / Limon were used to assess microcirculatory and regional circulation effects.
3. Changes in the cytokine pattern induced by esmolol administration in septic shock patients before administration of esmolol (H0) and 6 h after introduction of esmolol (H6).
4. Description of cardiac function during esmolol administration in septic shock patients. Echocardiography was used to assess ventricular function.

### Statistical analysis

Sample Size Calculation. The primary endpoint of this pilot study was the change in cardiac index between H0 and H6 in response to esmolol administration. A sample size of 25 patients would be required to detect a 0.6 SD change in cardiac index with a power of 80% and a 2-tailed significance level of 5%. Based on 31 patients in septic shock with a similar profile and hospitalized in our department, such a difference corresponds to a variation of 0.6 L/min/m^2^ in cardiac index, 2.5% in ejection fraction and 0.5 µg/kg/min in norepinephrine dosage.

Continuous variables are described as median (interquartile range) and categorical variables as frequencies (percentages). Values at H0 and at H6 as well as values at H0 and H7 were compared using the Wilcoxon signed-rank test for all continuous variables. For the three patients who prematurely discontinued the study treatment, values at H6 were replaced with the last value prior to treatment cessation and values at H7 were replaced with the first value after treatment cessation.

All analyses were performed using R statistical software (version 3.6.1, R Foundation for Statistical Computing, Vienna, Austria). The 2-tailed significance level was set at *p* < 0.05 with no adjustment for multiple comparisons.

## Results (Tables 1, 2, 3, 4 and 5, Fig. 1 and Additional file 1)

The study was terminated prior to enrollment completion due to a shift in ICU recruitment leading to a low inclusion rate. Nine consecutive stabilized tachycardic hyperkinetic septic shock patients treated with norepinephrine for a minimum of 6 h were included (Table 1). Esmolol was infused 9 (6.4–11.6) hours after norepinephrine introduction. Esmolol was ceased early in 3 out of 9 patients due to a marked increase in norepinephrine requirement associated with a picture of persistent cardiac failure at the lowest esmolol dose (Table 1: patients 7–9).

**Fig. 1:**
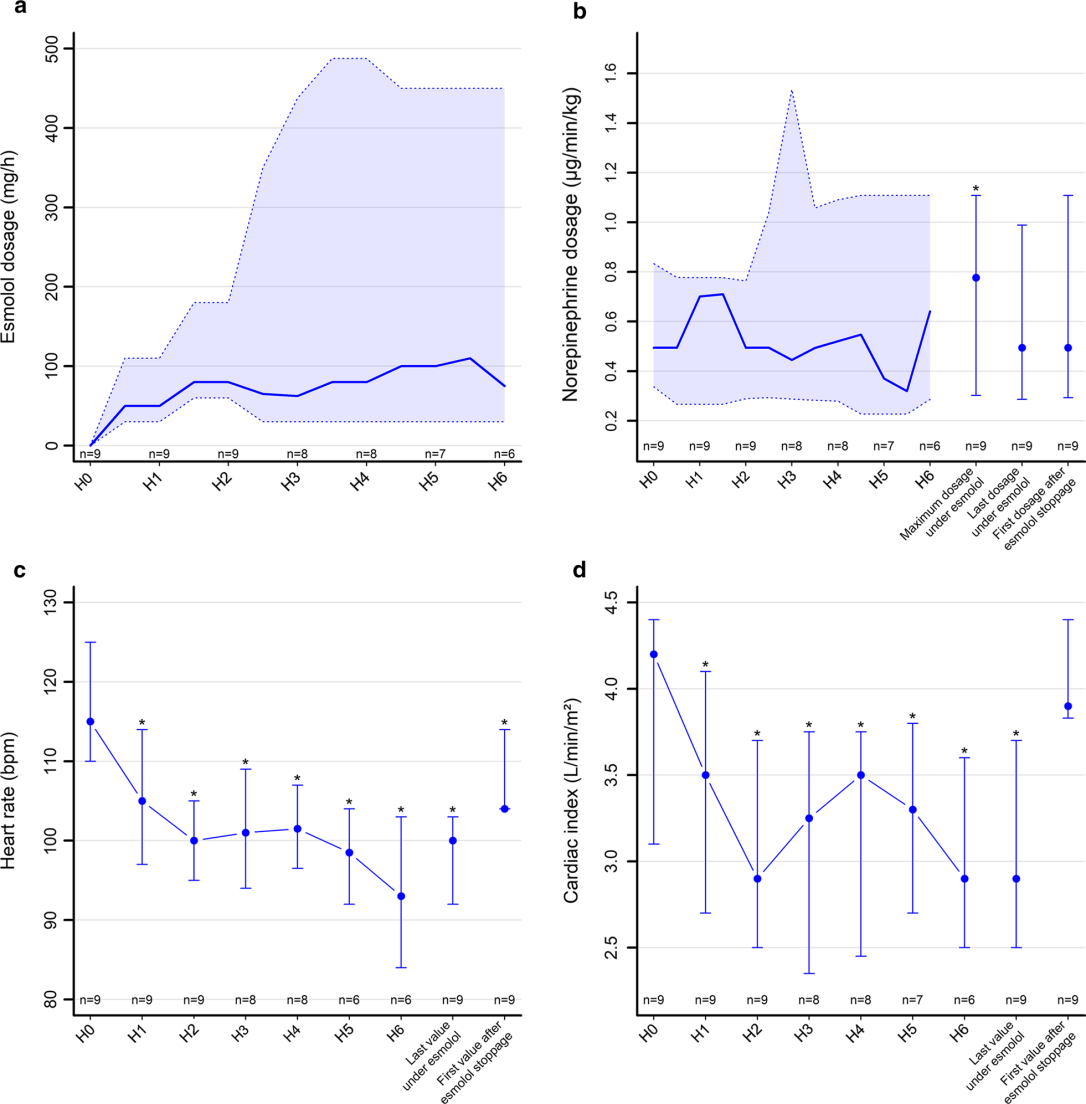
**a**, **b** Solid lines are median values while the shaded areas bordered by dotted lines represent the upper and lower quartiles. **c**, ** d** Points are median values, with the vertical bars indicating the interquartile range. The symbol (*) indicates a significant difference compared to H0 by the Wilcoxon signed-rank test (*p* < 0.05). M1 = maximum value under esmolol, M2 = last value under esmolol, M3 = first value after esmolol cessation

**Table 1:** Patient characteristics

Patient	Sex	Age	Sepsis etiology	SAPS II	SOFA	Norepinephrine HO µg/min/kg	Timing norepinephrine-esmolol introduction (hours)
1	F	82	Infectious pneumonia	79	12	2.00	8
2	H	58	Candidemia	61	14	0.57	9
3	H	54	Colo-pericardial fistulae	72	7	0.44	8
4	F	65	Infectious pneumonia	26	8	0.20	11
5	F	59	Ascitis	66	14	0.91	10
6	H	71	Soft skin	139	16	0.66	12
7	H	61	Infectious pneumonia	66	9	0,13	4
8	F	83	Biliary tract infection	73	14	0.83	5
9	F	24	Peritonitis	58	8	0.49	13

*SAPS II* Simplified Acute Physiology II Score, *SOFA* Sequential Organ Failure Assessment, *HO* O hours (baseline)

### Primary outcome (Fig. 1 and Table 2)

**Table 2:** Global hemodynamic and transpulmonary thermodilution parameters. Comparison H0–H6

	H0	H6	∆ H0–H6 (H6–H0)	*p* value**
Heart rate (bpm)	115 (110; 125)	100 (92; 103)	− 18 (− 25; − 15)	0.004
SAP (mmHg)	110 (102; 117)	103 (99; 104)	− 9 (− 14; 1)	0.16
DAP(mmHg)	54 (47; 55)	55 (54; 59)	4 (0; 8)	0.12
MAP (mmHg)	72 (68; 72)	72 (67; 75)	0 (− 3; 4)	0.52
Double product (mm Hg.bpm)	13,266 (12,138; 15,065)	9682 (8736; 10,609)	− 2657 (− 3305; − 2628)	0.008
CI(L/min/m^2^)	4.2 (3.1; 4.4)	2.9 (2.5; 3.7)	− 0.6 (− 1.2; − 0.5)	0.004
SVi (mL/m^2^)	29.8 (27.3; 37.8)	28.2 (27.2; 36.5)	− 0.2 (− 5.2; − 0.0)	0.25
CPI (W/m^2^	0.61 (0.54; 0.70)	0.48 (0.40; 0.58)	− 0.10 (− 0.17; − 0.06)	0.008
SVRi (dyn s m^2^ cm^−5^)	1164 (1143; 1412)	1379 (1333; 1876)	190 (139; 427)	0.004
CFI (1/min)	6.1 (4.8; 7.4)	3.7 (3.5; 4.8)	− 1.9 (− 2.6; − 1.0)	0.004
EPLW(mL/kg)	8 (7; 12)	9 (8; 11)	0 (− 1; 1)	0.91
CVP (mmHg)	8 (5; 8)	8 (6; 12)	1 (0; 2)	0.58
GEF (%)	20 (17; 21)	17 (15; 19)	− 3 (− 3; − 2)	0.004
Diuresis (mL/h)	100 (50; 150)	50 (10; 70)	− 60 (− 90; − 50)	0.016
SvcO_2_ (%)	73.6 (70.0; 87.0)	75.3 (71.0; 77.0)	− 3.5 (− 7.0; 3.5)	0.47
DO_2_i (ml/min/m^−2^)	444 (333; 615)	366 (319; 437)	− 68 (− 98; − 64)	0.031
VO_2_i (ml/min/m^−2^)	79 (61; 119)	93 (72; 96)	− 6 (− 13; 16)	0.84
Norepinephrine (µg/kg)	29.7 (20.2–50)	38.1 (18.1–66.5)	10.4 (5.7–25.5)	0.027

*HO* O hours (baseline), *H6* 6 h, *SAP* systolic arterial pressure, *DAP* diastolic arterial pressure, *MAP* mean arterial pressure, *Double product* SAP x HR, *CI* cardiac index, *SVi* indexed stroke volume, *CPI* cardiac power index, *SVRi* indexed systemic vascular resistances, *CFI* cardiac function index, *EPLW* extra pulmonary lung water, *CVP* central venous pressure, *GEF* global ejection fraction, *SvcO*_*2*_ central venous oxygen saturation, *DO*_*2*_*i* indexed oxygen delivery, *VO*_*2*_*i* indexed oxygen consumption, norepinephrine = hourly amount of norepinephrine

The use of esmolol was associated with a significant decrease in heart rate from 115 (110–125) to 100 (92–103) beats/min (*p* = 0.004), and a significant decrease in cardiac index from 4.2 (3.1–4.4) to 2.9 (2.5–3.7) l/min/m^−2^ (*p* = 0.004) without any change in either stroke volume or left ventricular end diastolic volume. Double product, a surrogate of myocardial oxygen consumption, also markedly decreased during esmolol infusion (*p* = 0.008). There was a trend towards a decrease in echocardiographic left ventricular ejection fraction (*p* = 0.074).

### Secondary outcome

For the global group, during esmolol infusion, the hourly amount of administered, norepinephrine significantly increased (Fig. 1; Table 2) (*p* < 0.05). ScVO_2_ and venous-arterial PCO_2_ gap remained unchanged (Table 2). Cardiac function index and global ejection fraction also significantly decreased (Table 2). Using echocardiography, systolic, diastolic as well as left and right ventricular function parameters significantly worsened (Table 3). After esmolol cessation, all parameters returned to baseline values.

**Table 3:** Echocardiographic parameters. Comparison H0–H6

	HO	H6	∆ H0–H6 (H6–H0)	*p* value
LVEF (%)	53 (50; 55)	45 (30; 57)	− 8 (− 18; 2)	0.074
LVEDV	89 (56; 114)	100 (44; 119)	9 (− 21; 43)	0.38
VTI (cm)	17 (15; 17)	14 (12; 16)	− 2 (− 3; − 1)	0.008
TDSa (cm/s)	11.0 (8.0; 13.0)	9.0 (7.0; 12.0)	− 1.0 (− 2.1; 0.0)	0.031
Peak E wave velocity (m/s)	0.90 (0.70; 1.00)	0.84 (0.80; 1.00)	0.00 (− 0.10; 0.12)	0.95
Peak E' wave velocity (m/s)	0.11 (0.09; 0.12)	0.09 (0.08; 0.10)	− 0.01 (− 0.03; − 0.01)	0.023
Peak A wave velocity (m/s)	0.77 (0.68; 1.03)	0.57 (0.47; 0.75)	− 0.21 (− 0.27; − 0.06)	0.016
E/A	0.91 (0.80; 1.33)	1.52 (0.97; 1.88)	0.47 (0.20; 0.62)	0.031
E/E’	7.6 (6.5; 9.0)	9.5 (8.4; 12.3)	2.6 (1.0; 3.3)	0.023
DTI S’ (cm/s)	9.0 (7.2; 14.0)	9.0 (8.0; 11.0)	− 1.0 (− 3.0; 0.0)	0.16
TAPSE (mm)	17.5 (16.0; 21.5)	15.5 (14.0; 16.5)	− 1.0 (− 4.0; − 0.5)	0.031

*HO* O hours (baseline), *H6* 6 h, *LVEF* left ventricular ejection fraction, *LVEDV* left ventricular end-diastolic volume, *VTI* velocity time integral, *TDSa* Tissue Doppler lateral mitral annulus peak systolic velocity, *E/A* early ventricular filling velocity to late ventricular filling velocity, *E/E′* mitral early diastolic velocity-to-early diastolic mitral annulus velocity, *DTI S′* derived tricuspid lateral annular systolic velocity S′ wave, *TAPSE* tricuspid annular plane systolic excursion

Oxygen delivery significantly decreased while oxygen consumption remained unchanged (Table 2)

Lactate (Table 4) and microcirculatory parameters (Table 5) did not change, while the majority of pro-inflammatory proteins decreased in all patients (Additional file 1, additional table).

**Table 4:** Arterial-venous gas parameters and lactate. Comparison H0–H6

	H0	H6	∆ H0–H6 (H6–H0)	*p* value
pH	7.34 (7.30; 7.39)	7.36 (7.31; 7.40)	0.01 (− 0.09; 0.02)	1.00
PaCO_2_ (mmHg)	34.4 (32.0; 36.0)	32.4 (26.2; 36.0)	− 3.3 (− 6.0; 6.4)	0.84
PvCO_2_ (mmHg)	37.4 (26.4; 42.0)	39.0 (36.5; 41.0)	− 0.9 (− 1.0; 2.9)	1.00
Delta PCO_2_ (mmHg)	6.0 (4.0;7.3)	6.0 (5.3;10.0)	4.0 (− 2.0; 6.3)	0.31
SaO_2_ (%)	95.9 (94.3; 98.0)	96.9 (94.2; 98.0)	0.5 (− 0.7; 1.0)	0.62
Lactate (mmol/L)	2.2 (1.5; 4.8)	2.4 (1.5; 4.5)	− 0.1 (− 0.3; 0.2)	0.69

*PaCO*_*2*_ normal arterial partial tension, *PvCO*_*2*_ mixed venous carbon dioxide tension, *Delta PCO*_*2*_ PvCO_2_–PaCO_2_, *SaO*_*2*_ oxygen saturation

**Table 5:** SDF, ICG clearance and NIRS parameters. Comparison H0–H6

	H0	H6	∆ H0–H6 (H6–H0)	*p* value
SDF				
Total vessel density	17.90 (14.88; 18.53)	16.98 (14.48; 19.56)	− 0.29 (− 0.82; 1.41)	1.00
Perfused vessel density	13.66 (11.34; 14.49)	14.51 (12.31; 15.91)	1.00 (− 1.26; 2.28)	0.69
Proportion of perfused vessel	67.41 (58.98; 75.55)	73.37 (66.26; 81.58)	3.96 (− 1.92; 13.83)	0.31
Microvascular flow index	1.96 (1.50; 2.44)	1.66 (1.33; 2.44)	− 0.08 (− 0.54; 0.00)	0.38
Plasma disappearance rate of indocyanine green				
Clearance rate (%/min)	10.8 (4.5; 17.0)	11.0 (4.7; 15.0)	0.2 (0.2; 0.5)	0.62
Retention rate at 15 min (%)	19.8 (6.9; 50.0)	19.0 (17.0; 49.4)	− 0.6 (− 0.8; 6.1)	0.81
Near-infrared spectroscopy				
StO_2_	74 (70; 85)	74 (72; 84)	1 (− 6; 2)	0.82
StO_2_ overshoot (%)	82 (76; 94)	84 (80; 90)	− 2 (− 3; 2)	0.84
StO_2_ desaturation slope (%/min)	− 6.6 (− 7.2; − 4.5)	− 6.9 (− 8.7; − 6.4)	− 0.2 (− 4.2; 0.1)	0.44
StO_2_ resaturation slope (%/s)	1.1 (0.8; 2.2)	1.2 (1.1; 1.7)	− 0.0 (− 0.2; 0.3)	1.00

*SDF* Sidestream dark field, *StO*_*2*_ tissue oxygen saturation

### Post-hoc analysis

Diuresis significantly decreased during esmolol infusion (*p* = 0.016).

## Subgroup analysis after excluding patients who did not complete the study (Additional file 1; additional table)

The evolution of heart rate, cardiac index, stroke, systemic vascular resistance volume, cardiac power index, cardiac function index, global ejection fraction, arterial-venous gases, lactate, SDF, ICG clearance and NIRS parameters remained similar. The evolution of the following parameters became non-statistically significant with a p value near 0.06: diuresis, VTI and TAPSE. The evolution of the hourly amount of required norepinephrine changed from a significant difference (*p* = 0.027) to a non-significant change (*p* = 0.094). Similarly, echocardiographic LVEF changed from a tendency to decrease (*p* = 0.074) to a non-significant change (*p* = 0.44).

## Discussion

The main results of the present study are that an early administration of esmolol in norepinephrine-treated hyperkinetic septic shock was associated with an increased risk of major hypotension and decreased cardiac index in one third of the patients. For the remaining patients, esmolol was associated with moderate hypotension necessitating an increase in norepinephrine and a depressed cardiac function without any effects on microcirculatory blood flow, lactate and ScVO_2_.

The main differences between the current study and the studies of Morelli et al. (6, 13) are the timing of esmolol infusion, the dose titration needed to achieve the predefined HR threshold which lasted 12 h in their study, with a first data collection point performed 24 h after esmolol initiation (thus 48 h after norepinephrine introduction versus 9 h in the present study), and a high use of levosimendan (49.3% versus 0% in our study). In the present study, esmolol administration had to be discontinued in one third of our septic shock patients due to a likely uncovered septic cardiomyopathy (14). Nevertheless, in the remaining patients, since stroke volume remained unchanged, the decrease in CI was predominantly related to the negative chronotropic effect of esmolol rather than a profound decrease in contractility. It is likely that the increase in norepinephrine doses participated in the increase in systemic vascular resistance index and concomitant decrease in cardiac index due to the resulting inhibition of the cardiac beta-1 receptors (15). Several important points should be discussed. First, in the present study, a fast esmolol titration was used in the early phase of septic shock. After reducing the heart rate, myocardial performance can differ significantly between patients depending on the relationship between their preload, ventricular filling and myocardial contractility status. The optimal combination of these components may therefore require a careful and slow titration and thus a longer time to safely achieve hemodynamic stability at a lower heart rate. Secondly, on the one hand, esmolol decreased cardiac index without increasing lactate levels or impairing microcirculatory parameters and appeared to be associated with certain anti-inflammatory effects. One the other hand, esmolol was associated with a decrease in diuresis and a marked cardiac impairment. Importantly, extravascular lung water did not increase suggesting adequate contractility, albeit reduced. This is in line with preserved SV and perfusion variables. This discrepancy might be explained (with the exception of patients 7–9) by sympathetic overstimulation leading to non-compensatory tachycardia and resulting, in turn, to an unnecessarily elevated cardiac index. Therefore, decreasing the latter was not accompanied by a decrease in tissue perfusion. This point is further strengthened by the observed decrease in oxygen delivery without any change in oxygen consumption arguing for an independent DO2/VO2 relationship. Finally, the very short half-life of esmolol in this indication allows a rapid reversibility of potential deleterious hemodynamics effects. Therefore, in a number of patients, elevated heart rate may be compensatory for decreased contractility rather than non-compensatory (sympathetic overstimulation), even in the later phases of septic shock (8).

The present study has several limitations. First, the study was terminated early due to a low enrolment rate. Second, patients were not randomized and therefore some of the observed effects could be due to spontaneous evolution although all of the parameters returned to baseline values after discontinuing esmolol treatment. Finally, 6 h is likely not sufficient to ascertain the consequences of non-hemodynamic effects of selective beta-1 blocking.

## Conclusion

In the very early phase of septic shock, heart rate reduction using a fast titration of esmolol is associated with an increased risk of hypotension and decreased cardiac index despite maintained adequate tissue perfusion.

## Supplementary Information

**Additional file 1**. H0–H6 comparison after excluding the three patients who discontinued esmolol prematurelyAdditional Table 6. Olink inflammation panel. H0–H6 comparison.

## Data Availability

All data generated or analyzed during this study are included in this published article. The data used to support the findings of this study are available from the corresponding author upon reasonable request.
