# Supplementary material for: Hemodynamic and anti-inflammatory effects of early esmolol use in hyperkinetic septic shock: a pilot study
Source: Crit Care. 2021 Jan 7;25:21. doi: 10.1186/s13054-020-03445-w (PMC7791811; doi:10.1186/s13054-020-03445-w)
Supplement: Supplementary file 1 — Additional file 1. H0–H6 comparison after excluding the three patients who discontinued esmolol prematurelyAdditional Table 6. Olink inflammation panel. H0–H6 comparison. [file 13054_2020_3445_MOESM1_ESM.docx]

**Additional Table . Olink inflammation panel. H0-H6 comparison**

|  | **H0** | **H6** | **∆ H0-H6**  **(H6 - H0)** | **p-value** |
| --- | --- | --- | --- | --- |
| IL8 | 10.42 (8.97 ; 13.71) | 8.78 (8.28 ; 12.87) | -0.68 (-0.83 ; -0.53) | **0.004** |
| VEGFA | 12.62 (12.15 ; 12.83) | 11.95 (11.72 ; 12.56) | -0.35 (-0.89 ; -0.19) | 0.074 |
| CD8A | 10.30 (10.07 ; 10.75) | 9.59 (9.15 ; 10.37) | -0.38 (-0.85 ; -0.10) | **0.027** |
| MCP-3 | 6.39 (6.06 ; 7.59) | 5.54 (5.12 ; 7.81) | -0.37 (-1.19 ; 0.15) | 0.13 |
| GDNF | 3.22 (2.41 ; 3.42) | 2.68 (2.24 ; 3.01) | -0.41 (-0.50 ; -0.30) | **0.012** |
| CDCP1 | 5.18 (4.73 ; 5.44) | 4.78 (4.14 ; 5.26) | -0.36 (-0.59 ; -0.15) | **0.020** |
| CD244 | 6.91 (6.39 ; 7.30) | 6.53 (5.86 ; 7.40) | -0.38 (-0.47 ; -0.28) | **0.012** |
| IL7 | 4.22 (3.71 ; 4.27) | 3.47 (3.08 ; 3.84) | -0.42 (-0.57 ; -0.37) | **0.004** |
| OPG | 12.42 (12.02 ; 12.46) | 12.29 (11.61 ; 12.88) | -0.29 (-0.47 ; -0.12) | 0.50 |
| LAP TGF-beta-1 | 8.36 (8.07 ; 8.97) | 7.99 (7.49 ; 8.71) | -0.37 (-0.42 ; -0.14) | **0.027** |
| uPA | 11.00 (10.48 ; 11.57) | 10.56 (9.51 ; 11.26) | -0.54 (-0.78 ; -0.18) | **0.012** |
| IL6 | 13.26 (12.38 ; 13.82) | 12.05 (9.99 ; 13.81) | -0.50 (-1.98 ; -0.37) | **0.012** |
| IL-17C | 3.94 (3.12 ; 6.13) | 3.76 (2.00 ; 5.68) | -0.63 (-0.76 ; -0.39) | **0.008** |
| MCP-1 | 14.64 (12.83 ; 14.89) | 13.31 (12.24 ; 14.10) | -0.68 (-1.34 ; -0.39) | **0.004** |
| IL-17A | 5.99 (5.13 ; 6.46) | 5.28 (4.10 ; 6.22) | -0.79 (-1.01 ; -0.17) | **0.039** |
| CXCL11 | 9.62 (8.01 ; 9.76) | 8.36 (7.33 ; 8.99) | -0.95 (-1.01 ; -0.58) | **0.004** |
| AXIN1 | 2.73 (1.84 ; 3.60) | 1.70 (1.11 ; 2.24) | -0.77 (-1.36 ; -0.57) | **0.004** |
| TRAIL | 6.80 (6.08 ; 7.22) | 6.58 (5.81 ; 6.99) | -0.33 (-0.64 ; -0.08) | **0.027** |
| IL-20RA | 1.46 (1.13 ; 1.58) | 1.48 (1.31 ; 1.65) | 0.04 (-0.16 ; 0.19) | 0.82 |
| CXCL9 | 9.57 (8.84 ; 10.68) | 8.58 (8.39 ; 10.25) | -0.52 (-1.03 ; -0.43) | **0.008** |
| CST5 | 5.79 (5.36 ; 6.34) | 5.30 (5.07 ; 5.95) | -0.40 (-0.49 ; -0.06) | 0.055 |
| IL-2RB | 1.20 (0.99 ; 1.31) | 1.16 (1.04 ; 1.25) | -0.04 (-0.16 ; 0.05) | 0.36 |
| IL-1 alpha | -0.77 (-0.90 ; -0.61) | -0.50 (-1.09 ; 0.27) | -0.06 (-0.48 ; 0.47) | 0.91 |
| OSM | 7.57 (6.93 ; 8.45) | 6.62 (6.43 ; 7.92) | -0.70 (-1.29 ; -0.49) | **0.004** |
| IL2 | 0.84 (0.62 ; 1.08) | 0.71 (0.69 ; 0.86) | -0.12 (-0.28 ; 0.06) | 0.16 |
| CXCL1 | 10.64 (9.34 ; 13.16) | 9.35 (8.52 ; 12.34) | -0.82 (-1.35 ; -0.55) | **0.004** |
| TSLP | 0.57 (-0.03 ; 0.81) | 0.46 (0.22 ; 0.65) | -0.13 (-0.49 ; 0.20) | 0.57 |
| CCL4 | 7.64 (6.61 ; 8.58) | 7.07 (6.12 ; 8.00) | -0.83 (-1.31 ; -0.34) | **0.004** |
| CD6 | 4.50 (3.98 ; 5.36) | 4.03 (3.48 ; 4.99) | -0.27 (-0.55 ; 0.18) | 0.16 |
| SCF | 7.60 (7.14 ; 8.21) | 7.31 (7.06 ; 7.46) | -0.27 (-0.62 ; -0.18) | **0.012** |
| IL18 | 9.43 (9.19 ; 10.47) | 9.19 (8.07 ; 10.34) | -0.49 (-0.55 ; -0.13) | 0.055 |
| SLAMF1 | 2.28 (1.98 ; 2.70) | 2.18 (1.77 ; 2.54) | -0.18 (-0.25 ; 0.03) | 0.055 |
| TGF-alpha | 5.82 (4.58 ; 6.88) | 5.11 (4.45 ; 6.12) | -0.38 (-0.71 ; -0.25) | **0.020** |
| MCP-4 | 12.06 (11.75 ; 13.51) | 11.31 (11.22 ; 12.36) | -0.47 (-1.16 ; -0.33) | **0.039** |
| CCL11 | 7.87 (6.49 ; 7.95) | 6.93 (6.14 ; 7.33) | -0.54 (-0.95 ; -0.17) | **0.020** |
| TNFSF14 | 6.87 (6.43 ; 7.34) | 6.42 (5.95 ; 6.92) | -0.41 (-0.64 ; -0.11) | **0.020** |
| FGF-23 | 9.09 (4.00 ; 10.63) | 10.05 (6.88 ; 10.54) | 0.34 (0.06 ; 0.96) | **0.027** |
| IL-10RA | 1.66 (1.06 ; 1.96) | 1.61 (1.14 ; 1.74) | -0.04 (-0.27 ; 0.00) | 0.13 |
| FGF-5 | 1.46 (1.14 ; 1.88) | 1.30 (1.17 ; 1.55) | 0.11 (-0.28 ; 0.12) | 0.43 |
| MMP-1 | 12.34 (12.19 ; 13.50) | 12.23 (10.77 ; 13.22) | -0.48 (-1.09 ; -0.28) | **0.004** |
| LIF-R | 4.30 (3.78 ; 4.75) | 3.92 (3.69 ; 4.70) | -0.10 (-0.28 ; -0.04) | 0.25 |
| FGF-21 | 11.58 (11.24 ; 12.04) | 11.95 (10.21 ; 12.04) | -0.41 (-0.75 ; 0.01) | 0.16 |
| CCL19 | 12.53 (12.24 ; 12.62) | 11.96 (11.72 ; 12.43) | -0.49 (-0.89 ; -0.28) | **0.004** |
| IL-15RA | 2.91 (2.02 ; 3.33) | 2.54 (1.76 ; 3.53) | -0.05 (-0.45 ; 0.08) | 0.50 |
| IL-10RB | 6.62 (6.41 ; 6.77) | 6.32 (6.00 ; 6.87) | -0.01 (-0.40 ; 0.10) | 0.50 |
| IL-22 RA1 | 2.17 (1.75 ; 3.05) | 2.23 (1.96 ; 2.79) | -0.03 (-0.21 ; 0.31) | 1.00 |
| IL-18R1 | 10.13 (9.43 ; 10.30) | 9.55 (9.36 ; 10.41) | -0.14 (-0.36 ; 0.13) | 0.36 |
| PD-L1 | 8.67 (7.64 ; 9.04) | 8.29 (6.94 ; 8.42) | -0.36 (-0.63 ; -0.25) | **0.027** |
| Beta-NGF | 1.76 (1.58 ; 1.82) | 1.45 (1.37 ; 1.61) | -0.21 (-0.31 ; 0.00) | 0.055 |
| CXCL5 | 10.88 (10.50 ; 12.05) | 9.44 (8.55 ; 10.57) | -1.06 (-2.22 ; -0.44) | **0.012** |
| TRANCE | 2.39 (1.91 ; 2.64) | 2.32 (1.87 ; 2.52) | -0.08 (-0.29 ; 0.28) | 0.91 |
| HGF | 11.76 (11.40 ; 13.65) | 11.38 (10.75 ; 13.01) | -0.32 (-0.83 ; -0.23) | **0.008** |
| IL-12B | 4.72 (4.57 ; 5.14) | 4.50 (4.17 ; 4.71) | -0.36 (-1.02 ; -0.01) | 0.098 |
| IL-24 | 4.58 (3.92 ; 6.56) | 4.29 (3.46 ; 6.63) | -0.01 (-0.85 ; 0.11) | 0.50 |
| IL13 | 0.72 (0.49 ; 0.91) | 0.47 (0.37 ; 0.74) | -0.17 (-0.18 ; -0.11) | **0.027** |
| ARTN | 1.15 (0.88 ; 2.02) | 0.94 (0.73 ; 1.34) | 0.03 (-0.45 ; 0.10) | 0.57 |
| MMP-10 | 10.64 (9.88 ; 11.29) | 10.44 (9.59 ; 11.35) | -0.24 (-0.40 ; -0.17) | **0.012** |
| IL10 | 7.30 (7.06 ; 8.13) | 6.96 (5.34 ; 7.44) | -1.02 (-1.89 ; -0.85) | **0.008** |
| TNF | 5.98 (4.54 ; 6.88) | 5.52 (4.60 ; 6.42) | -0.46 (-0.54 ; -0.06) | **0.020** |
| CCL23 | 12.48 (12.44 ; 12.58) | 12.35 (12.20 ; 12.42) | -0.15 (-0.21 ; -0.06) | 0.055 |
| CD5 | 5.32 (4.72 ; 5.89) | 5.52 (4.52 ; 5.72) | -0.17 (-0.40 ; 0.13) | 0.30 |
| CCL3 | 7.74 (5.92 ; 8.23) | 6.85 (5.24 ; 7.12) | -0.62 (-1.49 ; -0.13) | **0.008** |
| Flt3L | 9.01 (8.09 ; 10.39) | 7.83 (7.54 ; 9.46) | -0.38 (-0.93 ; -0.13) | 0.055 |
| CXCL6 | 9.67 (8.06 ; 10.58) | 8.17 (7.63 ; 10.13) | -0.47 (-0.63 ; -0.32) | **0.004** |
| CXCL10 | 13.27 (10.91 ; 13.55) | 11.68 (9.39 ; 13.41) | -0.69 (-1.38 ; -0.21) | **0.004** |
| 4E-BP1 | 10.03 (7.55 ; 10.27) | 7.99 (7.59 ; 9.82) | -0.40 (-1.18 ; -0.28) | 0.055 |
| IL-20 | 0.77 (0.70 ; 1.05) | 0.71 (0.55 ; 1.04) | -0.15 (-0.27 ; -0.05) | 0.20 |
| SIRT2 | 4.84 (3.78 ; 5.87) | 3.72 (3.22 ; 5.02) | -0.57 (-1.27 ; -0.23) | **0.004** |
| CCL28 | 2.39 (2.27 ; 3.34) | 2.50 (2.29 ; 3.03) | -0.03 (-0.19 ; 0.11) | 0.43 |
| DNER | 8.46 (8.07 ; 8.86) | 8.39 (7.47 ; 8.63) | -0.40 (-0.46 ; -0.08) | **0.027** |
| EN-RAGE | 4.46 (2.97 ; 5.96) | 4.56 (2.14 ; 6.21) | 0.16 (0.10 ; 0.25) | 0.57 |
| CD40 | 13.21 (11.70 ; 13.89) | 12.81 (11.68 ; 13.55) | -0.39 (-0.51 ; -0.06) | 0.13 |
| IL33 | 1.20 (1.06 ; 1.72) | 1.16 (1.04 ; 1.53) | -0.10 (-0.20 ; 0.15) | 0.50 |
| IFN-gamma | 8.94 (7.27 ; 9.29) | 7.22 (6.80 ; 8.00) | -1.08 (-1.60 ; -0.15) | **0.020** |
| FGF-19 | 9.39 (8.65 ; 10.64) | 8.34 (7.65 ; 10.56) | -0.32 (-0.70 ; -0.27) | 0.074 |
| IL4 | 0.86 (0.47 ; 0.88) | 0.59 (0.26 ; 0.76) | -0.07 (-0.20 ; -0.03) | 0.25 |
| LIF | 4.42 (3.22 ; 6.43) | 3.90 (2.89 ; 5.33) | -1.13 (-1.46 ; -0.38) | 0.074 |
| NRTN | 1.13 (1.08 ; 1.62) | 1.18 (0.91 ; 1.48) | -0.18 (-0.27 ; -0.03) | 0.13 |
| MCP-2 | 9.37 (8.68 ; 11.42) | 8.69 (7.23 ; 11.48) | -0.67 (-1.34 ; -0.35) | **0.008** |
| CASP-8 | 2.89 (2.79 ; 4.12) | 2.26 (2.00 ; 3.61) | -0.47 (-0.60 ; -0.35) | **0.020** |
| CCL25 | 5.94 (5.20 ; 6.39) | 5.63 (4.62 ; 6.23) | -0.16 (-0.35 ; 0.01) | 0.16 |
| CX3CL1 | 6.60 (5.51 ; 7.18) | 6.40 (5.07 ; 6.86) | -0.29 (-0.47 ; -0.05) | 0.074 |
| TNFRSF9 | 7.88 (6.95 ; 9.72) | 7.47 (6.63 ; 8.89) | -0.49 (-0.56 ; -0.32) | **0.020** |
| NT-3 | 2.42 (1.60 ; 2.50) | 2.51 (1.92 ; 2.65) | 0.07 (-0.37 ; 0.43) | 0.73 |
| TWEAK | 8.35 (8.32 ; 9.50) | 8.07 (7.50 ; 8.55) | -0.47 (-0.91 ; -0.31) | **0.008** |
| CCL20 | 12.12 (11.89 ; 12.89) | 11.73 (11.37 ; 12.56) | -0.32 (-0.44 ; -0.17) | 0.098 |
| ST1A1 | 2.09 (1.63 ; 3.08) | 2.38 (2.16 ; 3.17) | 0.09 (-0.46 ; 0.54) | 0.91 |
| STAMPB | 5.13 (3.97 ; 6.27) | 4.27 (3.50 ; 5.39) | -0.66 (-1.29 ; -0.33) | **0.008** |
| IL5 | 0.87 (0.84 ; 1.52) | 0.70 (0.62 ; 1.23) | -0.29 (-0.44 ; -0.20) | **0.027** |
| ADA | 5.58 (5.20 ; 6.53) | 5.63 (5.26 ; 6.26) | -0.08 (-0.20 ; 0.06) | 0.30 |
| TNFB | 3.33 (2.72 ; 3.85) | 3.21 (2.65 ; 3.55) | -0.13 (-0.31 ; 0.04) | 0.30 |
| CSF-1 | 10.53 (10.46 ; 10.68) | 10.49 (10.39 ; 10.57) | -0.09 (-0.23 ; -0.01) | **0.039** |

**Additional table: H0-H6 comparison after excluding the three patients who discontinued esmolol prematurely**

|  | **H0** | **H6** | **∆ H0-H6**  **(H6 - H0)** | **p-value** |
| --- | --- | --- | --- | --- |
| **Global hemodynamic and**  **transpulmonary thermodilution**  **parameters** |  |  |  |  |
| Heart rate (bpm) | 110 (110 ; 115) | 93 (84 ; 103) | -20 (-31 ; -17) | **0.031** |
| SAP (mmHg) | 110 (100 ; 131) | 103 (99 ; 103) | -12 (-27 ; -3) | 0.094 |
| DAP(mmHg) | 50 (46 ; 56) | 54 (49 ; 59) | 3 (-2 ; 8) | 0.34 |
| MAP (mmHg) | 69 (65 ; 72) | 70 (66 ; 75) | -2 (-4 ; 4) | 1.00 |
| Double product (mm Hg.bpm) | 13126 (11000 ; 15065) | 9209 (8372 ; 10609) | -2981 (-6329 ; -2628) | **0.031** |
| CI(L/min/m2) | 3.6 (3.1 ; 4.3) | 2.9 (2.5 ; 3.6) | -0.6 (-0.7 ; -0.5) | **0.031** |
| SVi (mL/m²) | 33.6 (28.2 ; 37.8) | 28.9 (28.2 ; 38.3) | -0.1 (-0.2 ; 0.5) | 0.84 |
| CPI (W/m²) | 0.57 (0.49 ; 0.65) | 0.46 (0.40 ; 0.52) | -0.09 (-0.14 ; -0.06) | **0.031** |
| SVRi (dyn.s.m².cm^-5^) | 1261 (1143 ; 1653) | 1586 (1333 ; 1876) | 167 (139 ; 427) | **0.031** |
| CFI (1/min) | 5.2 (4.5 ; 7.4) | 3.6 (3.0 ; 4.8) | -1.5 (-2.6 ; -1.0) | **0.031** |
| EPLW(mL/kg) | 10 (7 ; 16) | 10 (8 ; 14) | -0 (-3 ; 1) | 0.50 |
| CVP (mmHg) | 7 (5 ; 8) | 9 (7 ; 12) | 1 (0 ; 2) | 0.50 |
| GEF (%) | 18 (16 ; 24) | 16 (11 ; 22) | -2 (-3 ; -2) | **0.031** |
| Diuresis (mL/h) | 100 (50 ; 200) | 48 (10 ; 70) | -70 (-130 ; -50) | 0.062 |
| SvcO_2_ (%) | 73.6 (70.0 ; 87.0) | 75.3 (71.0 ; 77.0) | -3.5 (-7.0 ; 3.5) | 0.47 |
| DO_2_i (ml/min/m-^2^) | 444 (333 ; 615) | 366 (319 ; 437) | -68 (-98 ; -64) | **0.031** |
| VO_2_i (ml/min/m-^2^) | 79 (61 ; 119) | 93 (72 ; 96) | -6 (-13 ; 16) | 0.84 |
| Norepinephrine (µg/kg) | 33.2 (20.2 - 55.1) | 48.8 (18.1 - 147.3) | 18.2 (5.4 - 26.7) | 0.094 |
| **Echocardiographic parameters** |  |  |  |  |
| LVEF (%) | 53 (50 ; 58) | 51 (45 ; 60) | -3 (-18 ; 2) | 0.44 |
| LVEDV | 80 (56 ; 92) | 100 (44 ; 119) | 20 (-21 ; 43) | 0.31 |
| VTI (cm) | 16 (15 ; 20) | 14 (10 ; 18) | -2 (-4 ; -1) | 0.062 |
| TDSa (cm/s) | 10.5 (8.0 ; 15.0) | 8.0 (7.0 ; 12.0) | -1.5 (-4.0 ; 0.0) | 0.12 |
| Peak E wave velocity (m/s) | 0.81 (0.68 ; 0.92) | 0.80 (0.70 ; 1.11) | 0.00 (-0.10 ; 0.12) | 0.84 |
| Peak E' wave velocity (m/s) | 0.10 (0.09 ; 0.12) | 0.09 (0.07 ; 0.10) | -0.01 (-0.03 ; -0.01) | 0.062 |
| Peak A wave velocity (m/s) | 0.74 (0.70 ; 0.80) | 0.50 (0.50 ; 0.65) | -0.24 (-0.30 ; -0.08) | 0.062 |
| E/A | 0.92 (0.90 ; 1.04) | 1.60 (1.44 ; 1.66) | 0.62 (0.46 ; 0.63) | 0.12 |
| E/E’ | 8.0 (6.5 ; 9.0) | 10.4 (8.0 ; 12.3) | 3.2 (1.0 ; 3.5) | 0.094 |
| DTI S’ (cm/s) | 13.0 (7.2 ; 17.0) | 10.0 (8.0 ; 12.0) | -2.6 (-6.0 ; -1.0) | 0.22 |
| TAPSE (mm) | 18.0 (17.0 ; 20.0) | 16.0 (15.0 ; 16.0) | -1.0 (-5.0 ; -1.0) | 0.062 |
| **Arterial-venous gas parameters**  **and lactate** |  |  |  |  |
| pH | 7.34 (7.30 ; 7.39) | 7.36 (7.31 ; 7.40) | 0.01 (-0.09 ; 0.02) | 1.00 |
| PaCO_2_ (mmHg) | 34.4 (32.0 ; 36.0) | 32.4 (26.2 ; 36.0) | -3.3 (-6.0 ; 6.4) | 0.84 |
| PvCO_2_ (mmHg) | 37.4 (26.4 ; 42.0) | 39.0 (36.5 ; 41.0) | -0.9 (-1.0 ; 2.9) | 1.00 |
| Delta PCO_2_ (mmHg) | 6.0 (4.0 ; 7.3) | 6.0 (5.3 ; 10.0) | 4.0 (-2.0 ; 6.3) | 0.31 |
| SaO_2_ (%) | 95.9 (94.3 ; 98.0) | 96.9 (94.2 ; 98.0) | 0.5 (-0.7 ; 1.0) | 0.62 |
| Lactate (mmol/L) | 2.2 (1.5 ; 4.8) | 2.4 (1.5 ; 4.5) | -0.1 (-0.3 ; 0.2) | 0.69 |
| Sidestream dark field |  |  |  |  |
| Total vessel density | 17.90 (14.88 ; 18.53) | 16.98 (14.48 ; 19.56) | -0.29 (-0.82 ; 1.41) | 1.00 |
| Perfused vessel density | 13.66 (11.34 ; 14.49) | 14.51 (12.31 ; 15.91) | 1.00 (-1.26 ; 2.28) | 0.69 |
| Proportion of perfused vessel | 67.41 (58.98 ; 75.55) | 73.37 (66.26 ; 81.58) | 3.96 (-1.92 ; 13.83) | 0.31 |
| Microvascular flow index | 1.96 (1.50 ; 2.44) | 1.66 (1.33 ; 2.44) | -0.08 (-0.54 ; 0.00) | 0.38 |
| **Plasma disappearance rate**  **of indocyanine green** |  |  |  |  |
| Clearance rate (%/min) | 10.8 (4.5 ; 17.0) | 11.0 (4.7 ; 15.0) | 0.2 (0.2 ; 0.5) | 0.62 |
| Retention rate at 15 min (%) | 19.8 (6.9 ; 50.0) | 19.0 (17.0 ; 49.4) | -0.6 (-0.8 ; 6.1) | 0.81 |
| **NIRS** |  |  |  |  |
| StO_2_ | 72 (70 ; 81) | 74 (72 ; 84) | 2 (-6 ; 2) | 1.00 |
| StO2 overshoot (%) | 80 (72 ; 83) | 82 (78 ; 85) | 0 (-2 ; 3) | 0.88 |
| StO_2_ desaturation slope (%/min) | -6.6 (-7.2 ; -4.5) | -6.9 (-8.7 ; -6.4) | -0.2 (-4.2 ; 0.1) | 0.44 |
| StO_2_ resaturation slope (%/s) | 1.1 (0.8 ; 2.2) | 1.2 (1.1 ; 1.7) | -0.0 (-0.2 ; 0.3) | 1.00 |

**Abbreviations** : HO: O hours (baseline), H6: 6 hours, SAP: systolic arterial pressure, DAP: diastolic arterial pressure, MAP: mean arterial pressure, Double product : SAP x HR; CI: cardiac index, SVi: indexed stroke volume, CPI: cardiac power index, SVRi: indexed systemic vascular resistances, CFI: cardiac function index, EPLW: extra pulmonary lung water, CVP: central venous pressure, GEF: global ejection fraction, SvcO_2_: central venous oxygen saturation, DO_2_i : indexed oxygen delivery, VO_2_i : indexed oxygen consumption, norepinephrine = hourly amount of norepinephrine

LVEF: left ventricular ejection fraction, LVEDV: left ventricular end-diastolic volume, VTI: velocity time integral, TDSa: Tissue Doppler lateral mitral annulus peak systolic velocity, E/A: early ventricular filling velocity to late ventricular filling velocity, E/E': mitral early diastolic velocity-to-early diastolic mitral annulus velocity, DTI S’: derived tricuspid lateral annular systolic velocity S’ wave, TAPSE: tricuspid annular plane systolic excursion

PaCO_2_: normal arterial partial tension, PvCO_2_: mixed venous carbon dioxide tension, Delta PCO_2_: PvCO_2_ – PaCO_2_, SaO_2_: oxygen saturation

SDF: Sidestream dark field, StO_2_: tissue oxygen saturation
